# Engaging Sexual and Gender Minority Youth in HIV Interventions Through Gay Dating Apps: Recruitment Protocol

**DOI:** 10.2196/28864

**Published:** 2021-06-22

**Authors:** Manuel A Ocasio, Maria Isabel Fernandez, Ja'Lon M Joseph, Roxana Rezai, ATN CARES Team

**Affiliations:** 1 Department of Pediatrics School of Medicine Tulane University New Orleans, LA United States; 2 College of Osteopathic Medicine Nova Southeastern University Fort Lauderdale, FL United States; 3 Department of Epidemiology Fielding School of Public Health University of California, Los Angeles Los Angeles, CA United States; 4 Department of Psychiatry University of California, Los Angeles Los Angeles, CA United States; 5 Los Angeles LGBT Center Los Angeles, CA United States; 6 UT Southwestern Medical Center Dallas, TX United States; 7 School of Medicine University of California, San Fransisco San Fransisco, CA United States; 8 Division of Infectious Diseases David Geffen School of Medicine University of California, Los Angeles Los Angeles, CA United States

**Keywords:** HIV/AIDS, adolescents, recruitment, dating apps, msm, mHealth, gender, gay, behavior interventions, mobile phone

## Abstract

**Background:**

HIV continues to disproportionately impact sexual and gender minority youth (SGMY) in the United States. Public health efforts have increasingly focused on developing efficacious interventions to curb the spread of HIV among SGMY and help those living with HIV achieve and sustain viral suppression. However, recruiting and engaging SGMY in prevention and care interventions is challenging.

**Objective:**

During the past decade, gay dating apps have quickly emerged as popular web-based spaces in which SGMY congregate. Although the recruitment of SGMY through these apps has been commonly reported, advertisement is the typical modality used, and direct recruitment approaches are not adequately described. This study aims to describe the process for developing a direct recruitment protocol for use in gay dating apps.

**Methods:**

The Adolescent Medicine Trials Network Comprehensive Adolescent Research and Engagement Studies is a community-based research program consisting of 3 interrelated studies testing scalable behavioral interventions to improve HIV prevention and care engagement among youth aged 12-24 years in Los Angeles and New Orleans. To supplement our in-person recruitment approaches for Comprehensive Adolescent Research and Engagement Studies, the New Orleans site formed a gay dating app recruitment team. In April 2018, the team developed a loosely structured protocol that included study-specific profiles and sample language to guide initial recruitment efforts. Two self-identified Black, gay cisgender male field recruiters field-tested the protocol on the popular gay dating app Jack’d. During the field test, the recruitment team met weekly to discuss the recruiters’ experiences and user reactions. For example, we learned the importance of addressing concerns about study legitimacy and identifying appropriate ways to describe the study. We iteratively incorporated these lessons learned into the final protocol and developed a training program and tracking procedures before moving to full-scale implementation at both sites.

**Results:**

Adhering to this protocol yielded 162 enrollments in New Orleans (332 total enrollments across the two sites) throughout the recruitment period (April 2018 to August 2019). Most of these participants were sexual minority cisgender males (91%), and the remainder were identified as members of gender minority groups. We outlined step-by-step instructions on training staff, engaging users, and scheduling and tracking recruitment activities.

**Conclusions:**

This paper provides a practical guide for researchers and community-based providers to implement a gay dating app recruitment protocol. Our experience indicates that gay dating app recruitment is feasible and fruitful when the staff members are knowledgeable, flexible, honest, and respectful to the user. Perhaps the most salient lesson we learned in approaching gay dating app users is the importance of setting clear and transparent intentions without judgment. As gay dating apps continue to increase in popularity, researchers need to stay vigilant to changing formats and develop systematic approaches to harness their potential as invaluable recruitment strategies for SGMY.

**International Registered Report Identifier (IRRID):**

RR1-10.2196/28864

## Introduction

In the United States, sexual (ie, gay, bisexual, nonheterosexual identity, attraction, and behavior) and gender (ie, transgender, gender nonbinary, and gender identity incongruent with the sex assigned at birth) minority youth (SGMY), particularly youth of color, continue to be disproportionately affected by HIV. In 2018, 92% of all new HIV cases among youths aged 13-24 years were sexual minority cisgender males [1]. Although HIV data on gender minority youth are limited, young transgender women have been heavily impacted and have a high HIV prevalence [2]. For instance, current estimates suggest that 44% of Black or African American, 26% of Latina, and 7% of White transgender women live with HIV [3]. It is not surprising that public health efforts have increasingly focused on developing efficacious interventions to curb the spread of HIV among SGMY and help those living with HIV achieve and sustain viral suppression.

However, recruiting and engaging SGMY in prevention and care interventions has been challenging. It has long been accepted that one of the most effective recruitment approaches is meeting participants where they congregate. In the late 90s and the early 2000s, community venues such as clubs and bars were fruitful places for recruiting SGMY. This era also saw the advent of the internet as an increasingly popular social space, in which a number of studies leveraged internet chatrooms to recruit members of sexual minority groups [4,5]. Owing to their widespread use as dating and socializing sites, geolocation social networking (GSN) apps, such as Grindr, Jack’d, Scruff, and other social networking platforms are rapidly becoming the dominant recruitment venues for SGMY. The location features of these apps allow users to connect with other users from a broad geographic area and identify those nearby and easily accessible for dates or casual sexual encounters. By identifying users in close proximity to research staff, the geolocation features of these apps can also be harnessed to facilitate targeted SGMY recruitment efforts.

During the past decade, researchers have utilized GSN apps in a variety of ways to recruit sexual and gender minority participants for HIV research. Many studies have relied on passive methods, such as advertising banners and popup ads or posting a study-specific profile with contact information [5-13]. Others have used more active approaches to engaging with GSN users for study recruitment by having research staff create profiles and initiate conversations with users [14-17]. However, we could not find a single study that adequately described the actual procedures or steps used to recruit participants directly. Given the relative success and increasing utility of GSN recruitment, researchers should provide a clear step-by-step description of their recruitment processes and suggestions for overcoming obstacles inherent in using these apps for recruitment purposes.

In this paper, we describe our process for developing a direct recruitment protocol for use in gay dating apps that we implemented for a cooperative program project called Comprehensive Adolescent Research and Engagement Studies (CARES), which is one of 3 program projects and a coordinating center that comprises the Adolescent Medicine Trials Network for HIV/AIDS Interventions (ATN; ClinicalTrials.gov NCT03134833) [18].

## Methods

### Study Description

CARES is a community-based research program consisting of 3 interrelated studies (ATN 147, ATN 148, and ATN 149) testing scalable behavioral interventions to improve HIV prevention and care engagement among youth aged 12-24 years in Los Angeles and New Orleans [19]. Although we initially developed the protocol outlined in this manuscript to recruit youth at risk for HIV into ATN 149 in New Orleans, it was later integrated into recruitment efforts in Los Angeles [20].

We recruited 1494 youths for ATN 149 from May 2017 to August 2019, of whom 1052 were SGMY. Eligible youth had to (1) be between 12 and 24 years of age, (2) test HIV seronegative on a fourth-generation Alere test, and (3) be at risk of acquiring HIV as determined by their responses on a brief behavioral risk factor screener. After providing written informed consent, participants completed a baseline behavioral assessment, were tested for HIV and sexually transmitted infections, and randomized to one of four technology-based interventions of varying intensity. Participants completed a behavioral assessment and were tested for HIV and sexually transmitted infections at 4-month intervals for 2 years.

### Venue-Based Recruitment Strategies

For the first year of the study, we used traditional venue-based recruitment directly approaching youth at youth-focused community venues and agencies and popular community events. Other methods we used included distributing palm cards, posting study flyers at targeted venues, agencies, and events, and obtaining referrals from providers and study participants. These efforts, though successful in identifying a number of youth who reported multiple HIV risk behaviors, did not yield sufficient numbers of SGMY to meet recruitment targets.

To bolster SGMY enrollment, we targeted Pride events, college Gender and Sexuality Alliance activities, Parents and Friends of Lesbians and Gays meetings, bars and clubs popular among SGMY, and events hosted by other SGMY-serving organizations. We encouraged referrals by incentivizing SGMY participants for each successfully enrolled referral from within their networks. Cognizant that these approaches were only reaching youth willing to engage in community events and frequent popular venues, we decided to recruit SGMY through social networking sites, particularly gay dating apps.

### Gay Dating App Recruitment Protocol Development

#### Initial Field Test

In April 2018**,** we formed a social media recruitment team in New Orleans comprising the project director, a Latinx gay cisgender male, and 2 self-identified Black, gay cisgender male field recruiters. The team decided to first target Jack’d, a social networking smartphone app that uses geospatial technology to connect users based on geographic location, because it is one of the most popular gay dating apps among Black or African American sexual minority cisgender males in the South [21]. The team developed a loosely structured protocol that included study-specific profiles and sample language to guide the initial recruitment efforts and met weekly to discuss experiences and iteratively modify the protocol as needed. To determine the most productive recruitment periods, the team developed weekly schedules that varied by time of day and day of the week and monitored the yield. We adjusted the recruiters’ schedule to accommodate recruitment during *nontraditional* work hours, especially during evenings and weekends. Once we gained experience recruiting via Jack’d, we followed the same procedures to recruit other social networking apps, including Scruff and Grindr, with similar results. We tried recruiting Adam4Adam but were immediately blocked from using the app. The initial field test lasted 6 weeks, and we kept a series of ongoing notes and memos describing the process, recruiters’ experiences, and user reactions.

#### Profiles

**Figure 1:**
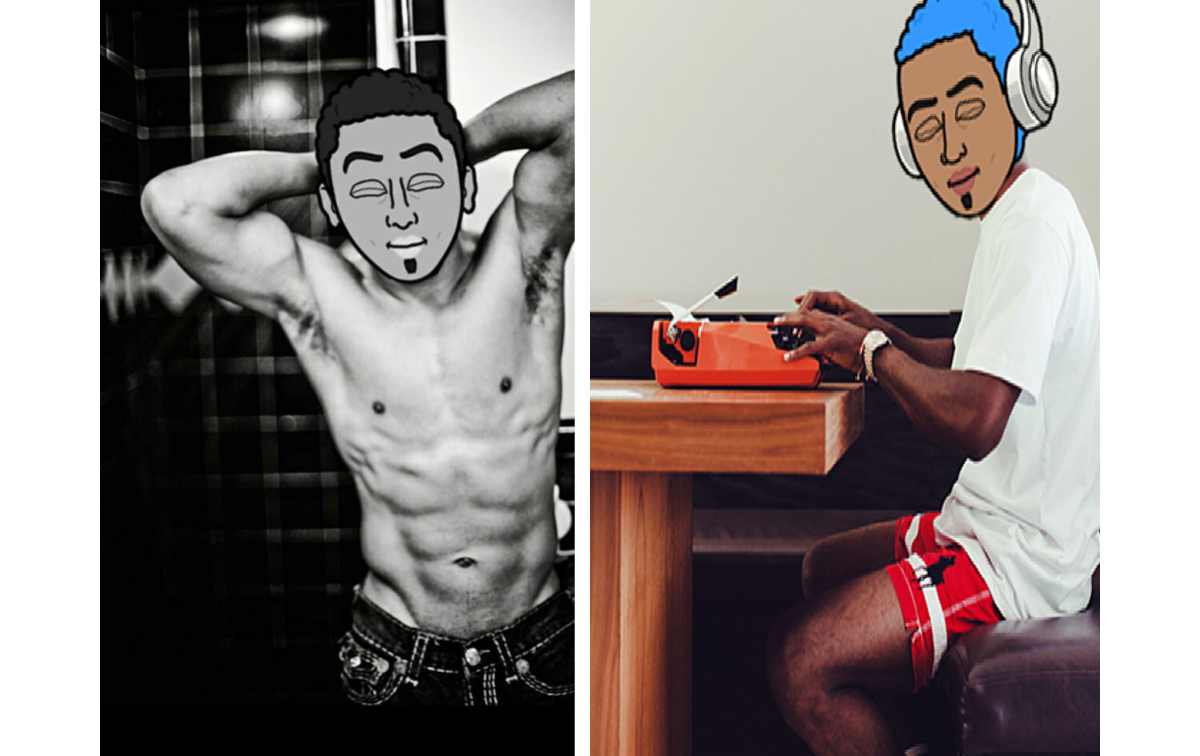
Sample profile photos.

Recruiters created their own study-specific profiles for use in apps. The characteristics of each profile reflected those common among app users. For example, we described ourselves as 20 or 21 years old; Black or African American man; versatile; and *looking for friends*, *networking*, *fun,* and other descriptors found in user profiles. Sample photos are illustrated in Figure 1. One profile used a bitmoji character superimposed on a photo of a field recruiter. This character, called *Nolan*, became our unofficial mascot that was created in response to the popularity of bitmojis. The other profile used a stock photo of a cisgender male who was described as attractive by the team.

#### Sample Language

We provided recruiters with scripted language to assist in initiating and maintaining conversations with users. Recruiters were encouraged to adapt the language as needed and share adaptations during our weekly meetings. Recruiters also kept ongoing records of the adaptations and user responses that we used to iteratively modify the suggested language, as shown in Textbox 1.

**Scripted language for conversations.**

**Introductions and Rapport Building**

- Hey wassup, I’m <NAME>. How are you doing tonight/today?
- What’s your name?
- It’s nice to meet you.
- Where are you from? I’m from <PLACE>.

**More Information About Participation**

- I work at [BLANK] for a community health program. We offer services to improve the sexual health of young guys under 24. Let me know if you’re interested and I can tell you more about it.
- I work for a health program at [BLANK] that is trying to help young guys under 24 to improve their sexual health. Your participation could be really helpful in finding ways to improve the health of our community. If you want to learn more, here is our website [INSERT LINK].
- It’s pretty simple. All you’d have to do is come in to get tested for sexually transmitted infections [& HIV] and answer a short survey about once every 4 months. You also learn about how to have fun, safe sex. You get paid every time you come in.
- The program goes on for 2 years and by the end of it, you stand to get a total of $350 over the whole program. We also offer free testing for HIV, syphilis, gonorrhea and chlamydia.

**Inappropriate Messages or Sexual Advances**

- I understand this app is used to hook up, which is OK. My goal is to connect you to information on our study and how to be involved. Click here for more info: [INSERT LINK].
- Have you read my bio? I’m on here to promote a study offering HIV & sexually transmitted infections testing. Is that something you would be interested in learning about?

#### Lessons From the Initial Field Test

After completing the initial field test, the entire New Orleans CARES team met weekly to review the findings, discuss recruiters’ experiences, and make further revisions to the protocol. In this section, we summarize the main challenges faced during the initial field test.

#### Repeated Interactions

When approached, some users said that they had previously been contacted by our recruiters, leading some to discontinue the conversations abruptly. Other users blocked our profiles because they had already been *hit up*. In reviewing these interactions, we realized that because our recruiters lived within 5 miles of each other, there was overlap in the users they could approach within their geospatial recruitment area. Although this is an artifact of GSN apps, it resulted in recruiters approaching the same users. We brainstormed strategies to minimize this possibility but quickly realized that documenting all of the recruiters’ user interactions would be a formidable, labor-intensive task for a number of reasons. First, users can change various aspects of their profiles (photos and text descriptions), making it difficult to track each user. Recruiters noted that this happened often. Second, the added effort necessary to document information on every user with whom the recruiters interacted would be time-consuming. We determined that the most viable approach when this occurred was to apologize and thank the user for their time.

#### Finding the Right Moment to Introduce the Study

We compared two approaches—stating intent upfront versus building rapport through casual conversations before introducing the study. Neither was perfect. At times, upfront intent leads to unresponsive users. When using the second approach, some users felt deceived or *catfished* given the time and *build-up* to mention the study. We opted to introduce the study as early as possible in the conversation to minimize perceptions of deceit.

#### Being Respectful and Following Users’ Wishes

We attribute part of our success in not being blocked from using the apps to our recruiters’ respectful tone, adherence to the wishes of the users, and understanding of the requirements of the app. For instance, recruiters did not send an excessive number of messages to multiple users within a given day. When a recruiter was *ghosted* (ie, abruptly discontinued a conversation), we did not immediately try to reconnect.

#### Inappropriate Messages or Sexual Advances

Given the nature of Jack’d, our recruiters were often contacted with sexually explicit photos or unwarranted sexual advances. Although we had anticipated and role-played some possible responses, some user reactions were unanticipated. For instance, a user asked our recruiter if, in addition to working on the study, they were also looking to *hook up*. When our recruiter responded, *only working!* the user was offended, said they felt judged, and blocked our profile. Reflecting upon the interaction, we concluded that the user might have perceived judgment from the use of an exclamation point. This heightens the importance of being mindful of subtle cues that users could misinterpret.

#### Establishing Legitimacy

One of our biggest challenges was convincing users that our recruiters were legitimate New Orleans’ CARES representatives. Although recruiters used scripted language to describe the study and sent a link to the ATN CARES website [22], some users remained skeptical and asked pointed questions without agreeing to be screened. Others discontinued the conversation but did not usually block our profile. This was surprising given that our website described CARES, linked to our Instagram account, and included information on community events we hosted and a contact form for additional information. To address these concerns, we made repeated attempts to revise the scripted language, which proved to be somewhat effective, and recruiters even sent pictures of their university ID and a team photo. We added an *Our Team* page to the website that included photos and bios from the team. Although these modifications were helpful, in rare instances, we continued to encounter users who questioned the study’s legitimacy.

#### Importance of Alternating Recruitment Periods

Throughout the initial field test, we varied the duration (2-5 h), day of the week, and time of each recruitment (5 PM to 2 AM) period. As anticipated, recruitment during *regular* workdays (9 AM to 5 PM) was not productive. The most fruitful recruitment periods, those that yielded the highest volume of engaging conversations with potential participants, spanned from Thursday evening through Sunday afternoon.

#### Scheduling Appointments

There was variation in the ease with which recruiters were successful in scheduling appointments. Recruiters urged interested users to schedule their screening and enrollment appointments as proximal to the time they were first engaged as possible. On rare and fortuitous occasions, users would ask to come in immediately, which we accommodated if feasible. However, it was more common for recruiters to communicate with users multiple days before securing an appointment. Many of these conversations would occur sporadically for multiple weeks or months before the users would commit to scheduling an enrollment visit. As recruiters often had dozens of conversations each week, we requested permission to *favorite* these users so we could easily reinitiate conversations rather than scrolling through the apps’ message log to find the user.

#### Other Considerations

Although a number of participants would provide their phone numbers to coordinate travel for their enrollment appointments, many would stay connected to the app until they walked through the door. The popularity of *Nolan*, our bitmoji profile photo, was noteworthy. Some potential participants asked for *Nolan* at their initial appointment, although they had been given the recruiter’s actual name.

#### Brainstorming in Real Time

As we could not anticipate all possible scenarios, recruiters would engage the project director and each other in real time when they faced *roadblocks* and were unsure how to respond, such as when users would make sexual advances or challenge the study’s legitimacy. Real-time brainstorming allowed recruiters to maintain user engagement and address their concerns seamlessly. It also improved recruiters’ abilities and skills in executing the protocol.

#### Protocol Refinement

When the initial field test was completed, we reviewed the field notes, discussed the recruiters’ experiences and user reactions, and used them to refine and finalize the protocol described in the *Results* section. We also developed a training program and tracking procedures before moving to full-scale implementation.

## Results

### Overview

In this section, we provide step-by-step instructions to guide researchers and community-based providers in implementing the gay dating app protocol we developed. Adhering to this protocol yielded 162 enrollments in New Orleans (332 total enrollments across the two sites) throughout the recruitment period (April 2018 to August 2019). Most of these participants were sexual minority cisgender males (91%), and the remainder were identified as members of gender minority groups.

### Step 1: Select Gay Dating Apps Popular With the Target Population

As the popularity of gay dating apps varies by region and target population, it is critical to identify the most popular apps used by the target population in the selected areas. With the aid of a community advisory board, key stakeholders, scientific literature, and research staff who are members of the target population, we developed a list of apps and cross-referenced the data to identify the most popular apps. Jack’d emerged as the most popular, and, with time, we expanded to other popular apps (Grindr and Scruff). Before making the final selections of apps, it is important to conduct an initial review of the terms of access and use governing each app to ensure that the research procedures align with the terms and conditions of the app. As the terms of use may change, it is a good idea to monitor the terms and conditions regularly. We recommend purchasing premium accounts rather than using the free version of the apps because the premium versions have expanded capabilities that maximize efficiency in recruitment, such as unlimited messaging and filtered searches (age, race or ethnicity, location, online or offline status, and HIV status). On the basis of our experience, our advice is to recruit on multiple apps, if possible. Not only does this allow different recruiters to target the same geographic area, but it also reduces the chances that recruiters approach the same user on the same day on the same app.

### Step 2: Train Staff

There are a number of concepts and skills that the staff members need to master to become effective recruiters on gay dating apps. In addition to having a solid command of the research protocol, including appointment scheduling and sample language, the staff members need to understand the guiding principles for effective interactions on the web and be proficient in using the lingo, symbols, and rapid exchanges typical of the medium. Furthermore, they must be cognizant, sensitive, and understanding of the sociocultural and contextual issues of the target population. Thus, the training program should incorporate didactic sessions, group discussions, modeling, and practice with coaching and feedback. A typical training period spans between 2 and 4 weeks, depending on the skill and experience of the staff. During the first few days of training, we focused on content and processes such as mastering the protocol, learning the lingo (eg, bareback and pnp), and gaining a deeper understanding of the target population. For the rest of the training period, we shifted toward skill acquisition and refinement through real-time observations and practice with coaching and feedback.

Although there are different approaches for gaining the requisite skills, after staff had mastered content and process, we paired an experienced social media recruiter with a trainee under the supervision of the project director. Initially, the trainee watched, took notes, and asked questions during recruitment events as the experienced recruiter engaged users in real time on the app. The trainees then engaged users on the apps with the guidance and supervision of the experienced recruiter. The team met regularly to discuss trainees’ progress, identify issues, and resolve emerging challenges. This process continued until the project director determined that the trainee had achieved competence in recruiting on their own. Our experience dictates that effective social media recruiters are not required to be members of sexual or gender minority groups but must be engaging, respectful, and willing to master the intricacies of the medium.

### Step 3: Create User Profile

Our young sexual minority cisgender recruiters were front and center in designing profiles that would appeal to potential participants. They were asked to create profiles with eye-catching photos and text descriptions that were easy to read and transparent in describing who we were and our intentions. These recruiters then met with the project director to discuss the profiles together and make refinements before initiating recruitment with the profile.

### Step 4: Develop and Manage Recruitment Efforts

To leverage the geospatial capabilities of the dating apps to recruit youth at the highest risk, we initially targeted zip codes with the highest HIV incidence among our target population and worked our way down to zip codes with fewer cases when the potential pool of users in an area was saturated. This was possible because the premium functionality of the apps allowed for searches at the zip code level and/or the ability to pinpoint an area on a map. We developed a schedule of recruitment periods that varied by time of day and day of the week, ensuring that we had maximal coverage across the periods. Although we had initially identified Thursdays through Sundays as being particularly high volume for user engagement, we wanted to make sure that we reached users who were active outside of those days and times. Thus, some shifts were scheduled as early as 7 AM and ended as late as 2 AM each day of the week. To ensure recruiters could balance their other study-related duties, recruitment shifts spanned from 2 to 4 hours and flexible work hours were instituted to accommodate their schedules. For instance, recruiters working late-night shifts were not expected to be at the office first thing the next morning.

At the end of each recruitment period, recruiters completed a tracking form that was used to monitor the yield of the recruitment period, identify patterns in engagement, and document the quantity and quality of the interactions. Specifically, recruiters tracked the number of (1) conversations in the shift, (2) scheduled appointments, (3) nonresponses, and (4) users engaged but who did not have an appointment scheduled. Recruiters could also add pertinent information about the conversations, such as things that worked well, challenges encountered, or any other information (Textbox 2). Although we tried to track conversations by users, we found this challenging because identifiable aspects of user profiles can be modified, including photos and text descriptions, and we could not devise a system to track communications with a large number of users, especially in ways that could be easily referenced by all recruiters.

**Sample of final dating app user engagement tracking sheet data points.**

**Label and Description**

- Time: interviewers added the date, start time, and end time of that shift.
- Location: the zip code that the interviewer used for their search within that given shift.
- Number of confirmed appointments: interviewers would enter the number of users with whom they were able to schedule an in-person study visit.
- Number of refusals: the number of users who asked not to be contacted anymore, blocked the interviewer, or explicitly stated disinterest in the study.
- Number ignored: the number of users to whom the interviewer sent a message but received no response.
- Number too far to participate: the number of users that were ineligible because not living in the area.
- Total number contacted: all contact attempts within the shift.
- Notes: interviewers add notable experiences (eg, not many age-eligible users and user questioned legitimacy).

### Step 5: Engage Users and Schedule Appointments

Although each recruiter developed their own unique engagement approach and script, being more free-flowing and less scripted in our conversations yielded the most promising results. Recruiters were free to use their own words, but constant copying and pasting from a predetermined script sounded too robotic and turned off users. Our most successful recruiters were those who kept the natural flow of the conversation while describing the study and motivating the user to schedule a screening appointment. Introducing the study earlier in the exchange rather than later was also important for some users not to feel deceived or led on. With some users, recruiters asked the user to read their profiles early in the rapport-building process. With other users, they wove the study description naturally into the conversation. After sending an introductory message, if recruiters encountered a minimally responsive user (long delays in responses) or a completely nonresponsive user, recruiters would move to directly describe the study and include their study phone number and email in the message. This would allow the user to make an informed decision about contacting the research team at a later time convenient for them. For users with whom conversations spanned multiple days or weeks, recruiters would ask to add the user as a *favorite* to easily reengage with them in the future. Being honest and respectful of the user at all times is essential, as it allows recruiters to follow the natural flow of the conversation and motivates the user to schedule a study appointment.

### Step 6: Hold Regular Meetings to Monitor Recruitment and Sustain Motivation

We held weekly meetings during which we used the tracking forms to review progress, discuss challenges, and revise the protocol and recruitment schedule. Recruiters shared their experiences and problem-solved difficult situations. If possible, we recommend having a senior recruiter on call to help resolve the most challenging issues in real time. We also made sure to celebrate recruitment successes and would encourage friendly competition between recruiters by acknowledging the recruiter who attained the most enrollments in a given month. This was key to building a mutually supportive environment and sustaining motivation.

## Discussion

This is one of the first studies to provide a detailed protocol for directly recruiting on gay dating apps. Our experience indicates that gay dating app recruitment is feasible and fruitful when the staff is knowledgeable, flexible, honest, and respectful to the user. Although internet-based recruitment has been commonly reported by many HIV researchers, advertisements are the typical modality used [5-11]. The recruitment procedures have not been well described in the few papers that discuss direct recruitment [14-17]. It is important to note that our protocol development process relied heavily on the expertise of our sexual minority cisgender male staff. Their voices were central in crafting the profile pictures and language and leading the training with other recruiters.

Contrary to what some might say, successful recruiters do not have to be members of sexual minority groups. Although the familiarity with navigating these apps is useful, it is not essential because these skills can be taught. Our structured training approach partnered trainees with experienced recruiters to provide first-hand exposure to the entire process and real-time coaching and feedback as they developed proficiency in navigating the app. We also found tremendous value in maintaining constant communication among the recruiters and the project director to collaboratively and instantaneously problem-solve issues that emerged.

Perhaps the most salient lesson we learned in approaching gay dating app users is the importance of setting clear and transparent intentions without judgment. As researchers, we must be aware of the impact our presence has on the spaces we enter. Virtual venues are perhaps the only spaces where many users, particularly those who are more isolated and stigmatized, connect and engage with others in their communities [23]. Gay dating apps allow users to connect on a variety of levels that can range from simple conversation to *hooking up*. We may be an unwelcome distraction and respect their time and intentions to use the app. Profiles and messages should be clear in describing who we are and our reasons for engaging with them. Furthermore, we must scrutinize the content of our communication and be mindful of the use of symbols and punctuation. For instance, users may misinterpret capitalization as screaming or a misplaced exclamation point as a judgment.

Although gay dating app recruitment was instrumental in helping us to meet our enrollment targets, it requires considerable time and effort and can be complicated when recruiters are engaged in multiple tasks. In our case, recruiters conducted other research activities, including participant assessments, retention, and follow-up visits. Balancing the demands of gay dating app recruitment with staff’s other study-related responsibilities requires ongoing monitoring and scheduling adjustments. Thus, both staff and management had to be flexible, adaptable, and willing to adjust their duties and responsibilities as needed. We recommend that researchers assign dedicated staff to gay dating app recruitment if feasible.

In recent years, gay dating apps have become more stringent in conditions for app usage, specifying that the apps are for private, noncommercial use. Currently, this may limit opportunities for direct recruitment methods for research, but app developers are becoming more interested in expanding their existing sexual health initiatives; thus, direct recruitment may be a possibility in the future. Grindr, for example, provides a comprehensive sexual health resource center and consistently has popup and banner ads for access to HIV prevention services (eg, pre-exposure prophylaxis and HIV testing) [24]. Our success with direct recruitment methods could indicate another avenue for connecting users to these resources. Furthermore, research has shown that young sexual minority males find direct communication and outreach of sexual health resources and services through gay dating apps acceptable [17]. New apps are continually being developed and may not impose similar restrictions on researchers.

In this manuscript, we described our experiences and provided step-by-step instructions that other researchers interested could follow and refine in recruiting through gay dating apps. This protocol can also be adapted by researchers engaging in social media recruitment in other types of apps. This protocol represents a first step that can be improved by targeted and rigorous research. For instance, studies examining different elements of profiles and pictures used by recruiters could yield data to improve engagement [25]. Qualitative studies to garner user perspectives and reactions to recruitment efforts could also be valuable. Using an implementation science framework could provide more insight into user experiences to refine communication approaches and tracking methods. Gay dating apps continue to increase in popularity. Researchers need to stay vigilant to changing formats and terms of service and develop systematic approaches to harness the potential of gay dating and other social networking apps as invaluable recruitment strategies for SGMY.

## Abbreviations

ATN: Adolescent Medicine Trials Network for HIV/AIDS Interventions
CARES: Comprehensive Adolescent Research and Engagement Studies
GSN: geolocation social networking
SGMY: sexual and gender minority youth

## References

[1] (2020). HIV and youth. Centers for Disease Control and Prevention.

[2] James SH, Rankin S, Keisling M, Mottet L, Anafi M (2016). The report of the 2015 U.S. transgender survey. National Center for Transgender Equality, Washington, DC.

[3] Becasen JS, Denard CL, Mullins MM, Higa DH, Sipe TA (2019). Estimating the prevalence of HIV and sexual behaviors among the US transgender population: a systematic review and meta-analysis, 2006–2017. Am J Public Health.

[4] Fernández MI, Warren JC, Varga LM, Prado G, Hernandez N, Bowen GS (2007). Cruising in cyber space: comparing internet chat room versus community venues for recruiting Hispanic men who have sex with men to participate in prevention studies. J Ethn Subst Abuse.

[5] Burrell ER, Pines HA, Robbie E, Coleman L, Murphy RD, Hess KL, Anton P, Gorbach PM (2012). Use of the location-based social networking application GRINDR as a recruitment tool in rectal microbicide development research. AIDS Behav.

[6] Iott BE, Veinot TC, Loveluck J, Kahle E, Golson L, Benton A (2018). Comparative analysis of recruitment strategies in a study of men who have sex with men (MSM) in Metropolitan Detroit. AIDS Behav.

[7] Phillips G, Grov C, Mustanski B (2015). Engagement in group sex among geosocial networking mobile application-using men who have sex with men. Sex Health.

[8] Goedel WC, Duncan DT (2015). Geosocial-networking app usage patterns of gay, bisexual, and other men who have sex with men: survey among users of grindr, a mobile dating app. JMIR Public Health Surveill.

[9] Huang E, Marlin RW, Young SD, Medline A, Klausner JD (2016). Using Grindr, a smartphone social-networking application, to increase HIV self-testing among black and Latino men who have sex with men in Los Angeles, 2014. AIDS Educ Prev.

[10] Rendina HJ, Jimenez RH, Grov C, Ventuneac A, Parsons JT (2014). Patterns of lifetime and recent HIV testing among men who have sex with men in New York City who use Grindr. AIDS Behav.

[11] Macapagal K, Coventry R, Puckett JA, Phillips G, Mustanski B (2016). Geosocial networking app use among men who have sex with men in serious romantic relationships. Arch Sex Behav.

[12] Sun CJ, Sutfin EL, Bachmann LH, Stowers J, Rhodes SD (2018). Comparing men who have sex with men and transgender women who use Grindr, other similar social and sexual networking apps, or no social and sexual networking apps: implications for recruitment and health promotion. J AIDS Clin Res.

[13] Martinez O, Wu E, Shultz AZ, Capote J, Rios JL, Sandfort T, Manusov J, Ovejero H, Carballo-Dieguez A, Chavez Baray S, Moya E, Matos JL, DelaCruz JJ, Remien RH, Rhodes SD (2014). Still a hard-to-reach population? Using social media to recruit Latino gay couples for an HIV intervention adaptation study. J Med Internet Res.

[14] Sun CJ, Stowers J, Miller C, Bachmann LH, Rhodes SD (2015). Acceptability and feasibility of using established geosocial and sexual networking mobile applications to promote HIV and STD testing among men who have sex with men. AIDS Behav.

[15] Winetrobe H, Rice E, Bauermeister J, Petering R, Holloway IW (2014). Associations of unprotected anal intercourse with Grindr-met partners among Grindr-using young men who have sex with men in Los Angeles. AIDS Care.

[16] Holloway IW (2015). Substance use homophily among geosocial networking application using gay, bisexual, and other men who have sex with men. Arch Sex Behav.

[17] Fields EL, Long A, Dangerfield DT, Morgan A, Uzzi M, Arrington-Sanders R, Jennings JM (2020). There's an app for that: using geosocial networking apps to access young black gay, bisexual, and other MSM at risk for HIV. Am J Health Promot.

[18] Lee S, Kapogiannis BG, Allison S (2019). Improving the youth HIV prevention and care continuums: the adolescent medicine trials network for HIV/AIDS nterventions. JMIR Res Protoc.

[19] Rotheram MJ, Fernandez MI, Lee S, Abdalian SE, Kozina L, Koussa M, Comulada WS, Klausner JD, Mayfield Arnold E, Ocasio MA, Swendeman D, Adolescent Medicine Trials Network (ATN) CARES Team (2019). Strategies to treat and prevent HIV in the United States for adolescents and young adults: protocol for a mixed-methods study. JMIR Res Protoc.

[20] Swendeman D, Arnold EM, Harris D, Fournier J, Comulada WS, Reback C, Koussa M, Ocasio M, Lee S, Kozina L, Fernández MI, Rotheram MJ, Adolescent Medicine Trials Network (ATN) CARES Team (2019). Text-messaging, online peer support group, and coaching strategies to optimize the HIV prevention continuum for youth: protocol for a randomized controlled trial. JMIR Res Protoc.

[21] Duncan DT, Park SH, Hambrick HR, Dangerfield Ii DT, Goedel WC, Brewer R, Mgbako O, Lindsey J, Regan SD, Hickson DA (2018). Characterizing geosocial-networking app use among young black men who have sex with men: a multi-city cross-sectional survey in the Southern United States. JMIR Mhealth Uhealth.

[22] (2021). ATN Cares.

[23] Garett R, Smith J, Chiu J, Young SD (2016). HIV/AIDS stigma among a sample of primarily African-American and Latino men who have sex with men social media users. AIDS Care.

[24] (2021). Grindr.

[25] Fernández MI, Varga LM, Perrino T, Collazo JB, Subiaul F, Rehbein A, Torres H, Castro M, Bowen G (2004). The internet as recruitment tool for HIV studies: viable strategy for reaching at-risk Hispanic MSM in Miami?. AIDS Care.

